# Host-Induced Gene Silencing of a G Protein α Subunit Gene *CsGpa1* Involved in Pathogen Appressoria Formation and Virulence Improves Tobacco Resistance to *Ciboria shiraiana*

**DOI:** 10.3390/jof7121053

**Published:** 2021-12-08

**Authors:** Panpan Zhu, Shuai Zhang, Ruolan Li, Changying Liu, Wei Fan, Tingzhang Hu, Aichun Zhao

**Affiliations:** 1 Key Laboratory of Biorheological Science and Technology, Ministry of Education, State and Local Joint Engineering Laboratory for Vascular Implants, Bioengineering College of Chongqing University, Chongqing University, Chongqing 400030, China; zppswgc@126.com; 2 The State Key Laboratory of Silkworm Genome Biology, College of Biotechnology, Southwest University, Chongqing 400715, China; 13243524615@163.com (S.Z.); lrl20000@163.com (R.L.); fanwei2034@163.com (W.F.); 3 Key Laboratory of Coarse Cereal Processing, Ministry of Agriculture and Rural Affairs, School of Food and Biological Engineering, Chengdu University, Chengdu 610106, China; lcyswu@163.com

**Keywords:** appressorium formation, *Ciboria shiraiana*, G protein α subunit, host-induced gene silencing (HIGS), mulberry, pathogenicity

## Abstract

Hypertrophy sorosis scleroteniosis caused by *Ciboria shiraiana* is the most devastating disease of mulberry fruit. However, few mulberry lines show any resistance to *C. shiraiana*. An increasing amount of research has shown that host-induced gene silencing (HIGS) is an effective strategy for enhancing plant tolerance to pathogens by silencing genes required for their pathogenicity. In this study, two G protein α subunit genes, *CsGPA1* and *CsGPA2*, were identified from *C. shiraiana.* Silencing *CsGPA1* and *CsGPA2* had no effect on hyphal growth but reduced the number of sclerotia and increased the single sclerotium weight. Moreover, silencing *CsGpa1* resulted in increased fungal resistance to osmotic and oxidative stresses. Compared with wild-type and empty vector strains, the number of appressoria was clearly lower in *CsGPA1-silenced* strains. Importantly, infection assays revealed that the virulence of *CsGPA1-silenced* strains was significantly reduced, which was accompanied by formation of fewer appressoria and decreased expression of several cAMP/PKA- or mitogen-activated protein-kinase-related genes. Additionally, transgenic *Nicotiana benthamiana* expressing double-stranded RNA targeted to *CsGpa1* through the HIGS method significantly improved resistance to *C. shiraiana*. Our results indicate that *CsGpa1* is an important regulator in appressoria formation and the pathogenicity of *C. shiraiana*. *CsGpa1* is an efficient target to improve tolerance to *C. shiraiana* using HIGS technology.

## 1. Introduction

Mulberry is an economically important tree with a long history in China [1]. Mulberry fruit have high nutritional value with abundant anthocyanin, flavone, resveratrol, and 1-deoxynojirimycin contents [2,3,4,5]. Mulberry leaves are the only feed for breeding silkworms, and have made great contributions to the prosperity of the Silk Road. Hypertrophy sorosis scleroteniosis caused by *Ciboria shiraiana* is the most devastating disease in mulberry fruit production, which causes huge economic losses in many mulberry growing areas every year, especially in southwest China [6,7]. The sclerotia of *C. shiraiana* fall to the ground along with the diseased mulberry fruit and remain in the soil over the winter. When the mulberry flowers bloom in spring, the sclerotia germinate and release a huge number of ascospores. The mature ascospores infect mulberry female flowers and eventually sclerotia are formed in the fruit, thereby completing its life cycle [8].

Sclerotia are dormant structures with an important role in the fungus life cycle. Sclerotia can maintain the viability of the pathogen for several years under an adverse environment. The sclerotia of *Sclerotinia sclerotiorum* can survive for up to 8 years and microsclerotia of *Verticillium dahliae* can survive for up to 15 years in soil [9,10]. Sclerotia of *C. shiraiana* can remain dormant in soil without a plant host for at least 2 years [11]. Therefore, effective control of sclerotia germination could be the key to reducing the losses caused by these pathogens.

The heterotrimeric G protein signaling pathway is conserved in eukaryotic organisms and plays important roles in sensing and responding to internal or external signals and various stresses [12]. The classical heterotrimer G protein consists of three subunits: α, β, and γ [12]. In the absence of an external signal, Gα and Gβγ subunits are bound into a G protein heterotrimer, which is in an inactive state. When receiving extracellular signal stimuli, G-protein-coupled receptors, as guanine nucleotide exchange factors, bind with the Gα subunit, resulting in conformational change and promoting an exchange of GDP to GTP on the Gα subunit and the dissociation of Gα subunit and Gβγ heterodimer [13]. Then, Gα and Gβγ activate downstream effectors, including adenylyl cyclases, mitogen-activated protein kinases (MAPK)s, ion channels, phosphodiesterases, and phospholipases [14,15,16,17]. Previous studies have shown that Gα subunits are involved in the regulation of various physiological processes of fungi [18,19,20]. In *Metarhizium robertsii*, *MrGpa1* deletion caused reductions in the number of conidia formed, germination, stress sensitivity, and pathogenicity [18]. In *Aspergillus fumigatus*, three Gα subunits were found and shown to participate in regulation of hyphae growth, asexual development, germination, oxidative stress tolerance, and gliotoxin production [19]. In addition, *Mga1* was demonstrated to play an important role in the regulation of hyphae growth, fungi reproduction, and the production of some secondary metabolites [20]. Furthermore, Gα3 coordinates with cAMP and BMP1 to regulate the penetration ability and conidia germination of *Botrytis cinerea* [21].

First discovered in plants, RNA silencing or RNA interference (RNAi) is post-transcriptional gene silencing and this mechanism is conserved in eukaryotes [22,23]. Subsequently, RNAi has been widely applied in gene function research in plants, animals, and fungi [24,25,26]. Recent studies have shown a novel mechanism of communication between parasites and their hosts, termed cross-kingdom RNAi, which involves small interference RNAs expressed in the host that target pathogen-virulence-related genes and can be delivered from plants to plants, pests, and fungi—a term known as host-induced gene silencing (HIGS) [27,28,29,30,31]. Previous studies have indicated that HIGS is a powerful tool to protect plants from a variety of pathogenic fungi infection, including wheat, banana, and lettuce [31,32,33].

Hypertrophy sorosis scleroteniosis caused by *C. shiraiana* is the most destructive fungal disease in the mulberry fruit industry [34]. The G protein α subunits were shown to be involved in fungal development, appressorium formation, and pathogenicity [13,18,20]. However, the function of the G protein α subunit in *C. shiraiana* development and pathogenicity remains unclear. In this study, we characterized the functions of two G protein α subunit genes from *C. shiraiana*, *CsGPA1* and *CsGPA2*, and investigated whether HIGS can be used to improve tobacco resistance to *C. shiraiana* by targeting the pathogenicity-related gene *CsGPA1* from *C. shiraiana*.

## 2. Materials and Methods

### 2.1. Plant and Fungal Materials and Growth Conditions

The previously fully genome-sequenced wild-type *C. shiraiana* strain WCCQ01 was used in this study [35]. Fungal strains were cultured on potato dextrose agar (PDA) plates at 25 °C. *Nicotiana benthamiana* plants were used and cultured in a light incubator at 25/18 °C and 16/8 h of light/dark, with a light intensity of 5000 lx.

### 2.2. Cloning and Bioinformatics Analysis of CsGPA1 and CsGPA2 Genes

The total RNA of *C. shiraiana* was extracted using TRIzol reagent according to the manufacturer’s procedures (Invitrogen, Carlsbad, CA, USA). A PrimeScript™ RT Reagent Kit (Takara Bio., Shiga, Japan) was used to synthesize cDNA. Base on the coding sequence obtained from the *C. shiraiana* gene database, primers were designed using Primer 5.0. The *CsGPA1* (GenBank accession number: MZ574567) and *CsGPA2* (GenBank accession number: MZ574568) genes were cloned from *C. shiraiana* cDNA using the primers *CsGPA1*-F and *CsGPA1*-R, and *CsGPA2*-F and *CsGPA2*-R, respectively (Table S1). The National Center for Biotechnology Information Blastp tool was used to search for homolog proteins from other fungi species (http://blast.ncbi.nlm.nih.gov/Blast.cgi, accessed on: 7 December 2021). The protein domain was predicted by SMART (http://smart.embl-heidelberg.de/, accessed on: 7 December 2021). Sequence alignment was performed using ClustalX software. The MEGA4 software using neighbor-joining method was applied to construct the phylogenetic tree.

### 2.3. Real-Time RT-PCR Analysis

To detect the expression of *CsGPA1* and *CsGPA2* in different organs, the hyphae, initial sclerotia, developing sclerotia, mature sclerotia, apothecia, and conidia of *C. shiraiana* were collected. Additionally, to analyze the expression of *CsGPA1* and *CsGPA2* genes during the infection process of *N. benthamiana*, fresh agar plugs of mycelia of the same size were inoculated onto tobacco leaves and the leaves were collected at time points of 0, 12, 24, 48, 72, and 96 h post-inoculation (hpi). Total RNA was extracted using TRIzol reagent and used to synthesize cDNA with a PrimeScript™ RT Reagent Kit. Real-time quantitative PCR (qRT-PCR) was performed using a SYBR Green Reagent Kit (Takara Bio.) for 40 cycles with a final extension for 10 min. The *β-tubulin* gene served as the internal reference and the relative expression levels were determined using the 2^–ΔΔCt^ method [36].

### 2.4. Construction of CsGPA1 and CsGPA2 Silencing Vectors and Fungi Transformation

The pSilent-1 was digested by the *Sac*I and *Xba*I and an approximately 1900 bp fragment (*PtrpC-Hphr-TtrpC*) was purified and then ligated into pCambia1300, which was digested by the same restriction enzymes to produce pCambia1300-PHT. Partial sense and anti-sense fragments of *CsGPA1* genes were amplified from *C. shiraiana* cDNA using the primers *SiCsGPA1*-F/R and *SiCsGPA1*-F1/R1 with specific restriction sites. The sense fragments of *CsGPA1* and *CsGPA2* were cloned into psilent-1 by *Xho*I/*Hind*III, then anti-sense fragments were also ligated into the pSilent-1 plasmid in succession by *Bgl*II/*Kpn*I. Subsequently, the plasmids were digested by the *Xba*I restriction enzyme and fragments containing the target genes were ligated with the linearized pCambia1300-PHT vector by T4 DNA Ligase (Takara Bio.) (Figure S1a). The *CsGPA1* and *CsGPA2* silencing vectors were transformed into *C. shiraiana* via the *Agrobacterium*-mediated transformation method as described by Yu et al. [37]. A monoclonal colony was transferred to PDA plates with 60 μg/mL hygromycin and selected for three generations in succession. The primers for the *hygromycin*-resistant gene (*Hyg*-F and *Hyg*-R) were used to verify the *CsGPA1*- and *CsGPA2*-silenced transformants.

### 2.5. Construction of HIGS Plasmids and Tobacco Transformation

Partial sense and anti-sense fragments of *CsGPA1* were amplified from *C. shiraiana* cDNA using the primers *dsCsGPA1*-F/R and *dsCsGPA1*-F1/R1 shown in Table S1. The fragment was blasted in *C. shiraiana* (GenBank accession number: VNFM00000000) and *N. benthamiana* (https://solgenomics.net/tools/blast/, accessed on: 7 December 2021) genome and no discernible homology to off-target sequences was found. Additionally, Si-Fi software (v21) was used for off-target prediction (http://labtools.ipk-gatersleben.de, accessed on: 7 December 2021) and no off-target hits were found. The sequenced fragments were ligated with pHANNIBAL in succession by *Xho*I/*Kpn*I and *Hind*III/*BamH*I. Then, the intermediate vector pHANNIBAL was digested with the restriction enzymes *Sac*I and *Spe*I, and the fragments containing the target genes were purified and inserted into the destination vector pBin19 linearized by *Sac*I and *Xba*I (*Spe*I and *Xba*I are a pair of isocaudarners) (Figure S1b). The HIGS plasmid was transformed into *Agrobacterium tumefaciens* strain LBA4404 using a chemical method. A leaf disk co-cultivation method was used for *N. benthamiana* transformation [38].

### 2.6. Morphological Characteristics and Phenotypic Analysis of RNAi Strains

Wild-type (WT), empty vector (EV), and RNAi strains were cultured on PDA medium at 25 °C in an incubator. Hyphal growth was measured and photographed at 24 and 36 h. Sclerotia development phenotypes were photographed at 14 days post inoculation (dpi). Meanwhile, numbers and mass of sclerotia were determined. The fresh agar plugs were cultured on a hydrophobic glass slide to induce appressoria formation. The appressoria were observed using bright field microscopy (Olympus, Tokyo, Japan). For pathogenicity assays, fresh agar plugs of control and RNAi strains were inoculated onto *N. benthamiana* leaves. Pictures were taken at 48 hpi and the lesion size was measured using ImageJ software.

### 2.7. Stress Adaptation Assay

We analyzed the effects of the downregulated expression of *CsGPA1* and *CsGPA2* on the sensitivity of *C. shiraiana* to different stresses, as described by Feng et al. [39]. Fresh agar plugs of WT, EV, and RNAi strains were inoculated on CM medium supplemented with osmotic stress agents 1 M NaCl and 1 M KCl, oxidative stress agent 5 mM H2O2, and cell wall disturbing agents 0.005% sodium dodecyl sulphate (SDS) and 300 μg/mL Congo Red (CR) [39]. Pictures were taken at 48 hpi and the colony diameters were measured. All treatments were replicated three times.

### 2.8. Extracellular Laccase and Peroxidase Activity Assays

Extracellular laccase and peroxidase activities were determined as described by Chi et al. with slight changes [40]. The mycelia inoculated in CM liquid cultures for 3 days were removed completely by filtration and centrifugation for 10 min at 5000× *g* and 4 °C. The reaction mixtures (1 mL) consisted of 50 mM acetate buffer (pH 5.0) and culture filtrate mixed with the 10 mM ABTS (200 mL). The peroxidase and laccase activities were respectively determined in reaction mixtures with or without 3 mM H_2_O_2_. Then, the reaction mixtures were incubated for 5 min at 25 °C in darkness and absorbance was determined at 420 nm.

### 2.9. Relative Biomass and Histological Observations of Fungi in HIGS Plants

The leaves of WT and transgenic plants after inoculation with WT strains for 48 hpi were collected and then quickly homogenized into powder in liquid nitrogen. Total RNA and DNA were isolated to determine the expression of *CsGPA1* and the relative biomass, respectively. The determination of relative biomass was performed according to Zhang et al. [41].

Fungal development in HIGS plants was observed as described by Redkar et al. [42]. Briefly, N. benthamiana leaves were collected at 10 hpi and then fixed with 100% ethanol and 10% KOH to remove chlorophyll. After this step, they were stained with Wheat Germ Agglutinin Alexa Fluor 488 (WGA-AF488) (Thermo Fisher Scientific, Waltham, MA, USA) as described by Redkar et al. [42]. All microscopy assays were performed using a laser scanning confocal microscope (Leica Microsystems, Wetzlar, Germany).

### 2.10. Statistical Analysis

All the data in this study were obtained from at least three independent repetitions. The final results are shown as means ± standard deviations (SD). SPSS Statistics 17.0 software (SPSS Inc., Chicago, IL, USA) was used for statistical analysis. The graphs were created using GraphPad Prism 5 software (GraphPad Software Inc., La Jolla, CA, USA).

## 3. Results

### 3.1. Identification and Expression Analysis of CsGPA1 and CsGPA2 Genes

A BLAST search using G protein α subunits from *Magnaporthe oryzae* and *Saccharomyces cerevisiae* as queries found two putative G protein α subunit genes in the *C. shiraiana* genome. The two genes were cloned from *C. shiraiana* cDNA and named *CsGPA1* and *CsGPA2*. The characteristics of the predicted G protein α subunit genes are shown in Figure S2. The full-length genomic sequences of *CsGPA1* and *CsGPA2* were 1341 and 1365 bp, respectively. The open reading frame lengths were 1011 and 1062 bp with six and five exons, respectively (Figure S2a). Both CsGPA1 and CsGPA2 were predicted to contain a G protein α subunit domain by SmartBLAST (Figure S2b). Phylogenetic analysis showed that CsGPA1 and CsGPA2 had the closest relationships with *S. sclerotiorum* and *B. cinerea*, which also belong to Sclerotiniaceae (Figure S2c).

**Figure 1:**
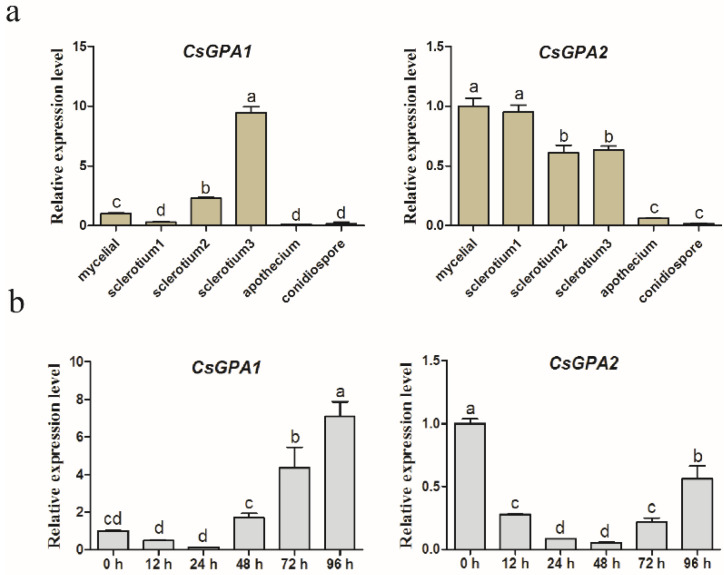
Relative expression levels of *CsGPA1* and *CsGPA2* in different tissues (hyphae; sclerotia1, initial sclerotia; sclerotia2, developing sclerotia; sclerotia3, mature sclerotia; apothecia; and conidia) (**a**) and during infection (**b**) by qRT-PCR. Significant differences (*p* < 0.05) among columns were detected using a one-way ANOVA followed by Duncan’s multiple range test and are labeled with different letters above the bars.

To analyze the expression patterns of *CsGPA1* and *CsGPA2* at various developmental stages and plant infection process, their relative expression levels were determined using qRT-PCR. Expression levels of *CsGPA1* and *CsGPA2* were induced during sclerotial development stages. The expression level of *CsGPA1* peaked at sclerotia 3 (mature sclerotia) and *CsGPA2* was mainly expressed in hyphae and sclerotia 1 (initial sclerotia), implying that *CsGPA1* and *CsGPA2* may participate in sclerotia development (Figure 1a). Both *CsGPA1* and *CsGPA2* showed upregulation in the late stage of plant infection (48–96 hpi). However, *CsGPA2* expression was significantly decreased during the infection process compared with 0 hpi. It should be noted that *CsGPA1* expression was lower in early-stage and higher in late-stage infection, indicating that it may play an important role in *C. shiraiana* pathogenicity (Figure 1b).

### 3.2. Characterization of CsGPA1- and CsGPA2-Silenced Strains

To determine the efficiency of *CsGPA1* and *CsGPA2* silencing, the candidate strains were screened by genomic PCR amplification of the hygromycin resistance gene (Figure S3a,b). Additionally, the relative expression levels were determined by qRT-PCR. The expression levels of target genes were significantly lower in RNAi strains compared with WT and EV, and target gene expressions of all these mutants were reduced below 50% of WT, except for *SiCsGPA2–5* (Figure S3c,d). In *CsGPA1*- and *CsGPA2*-silenced strains, three mutants with silencing efficiency of above 60% were selected for further studies.

**Figure 2:**
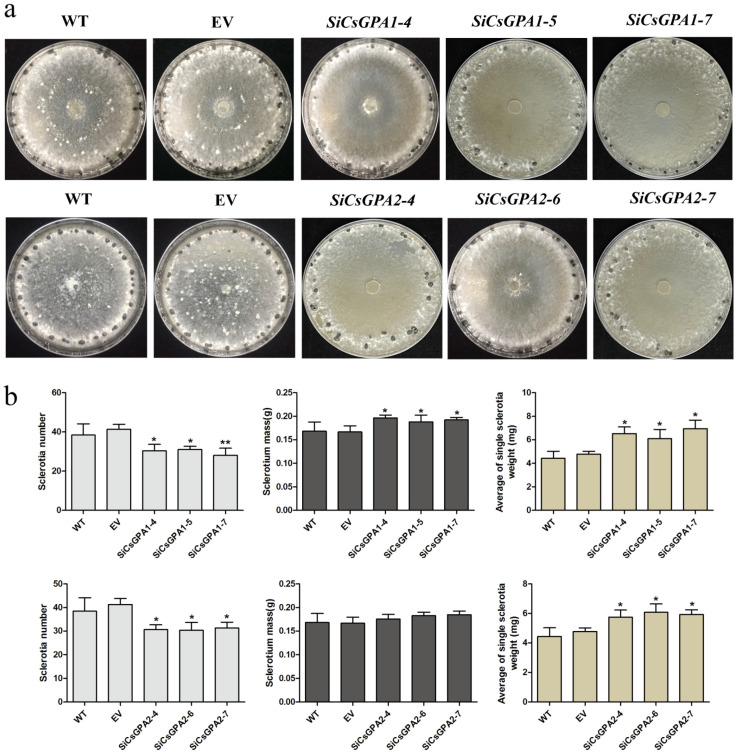
Development of sclerotia in wild-type (WT), empty vector (EV), and gene-silenced strains (*CsGPA1* and *CsGPA2*): (**a**) morphologies of sclerotia; (**b**) number, mass, and average of single weight of sclerotia formed at 15 dpi. Three strains were selected for assay and every strain was replicated three times. Scale bars correspond to 2 mm. The data represent the means ± SD of three independent replicates, and the differences between the mutants and the controls or EV were analyzed by one-way ANOVA followed by Duncan’s multiple range test. * *p* < 0.05, ** *p* < 0.01.

Hyphal growth rates of *CsGPA1*- and *CsGPA2*-silenced strains did not significantly differ from those of WT and EV strains (Figure S4). However, no microsclerotia were formed in *CsGPA1* and *CsGPA2* RNAi strains (Figure 2a). The numbers of sclerotia were significantly lower in *CsGPA1* and *CsGPA2* RNAi strains, and the average weight of single sclerotia increased compared with WT and EV (Figure 2b). *CsGPA1* RNAi strains had significantly higher total mass of sclerotia; however, there were only slight increases for *CsGPA2* RNAi strains (Figure 2b).

### 3.3. CsGPA1 Is Required for Compound Appressoria Formation

**Figure 3:**
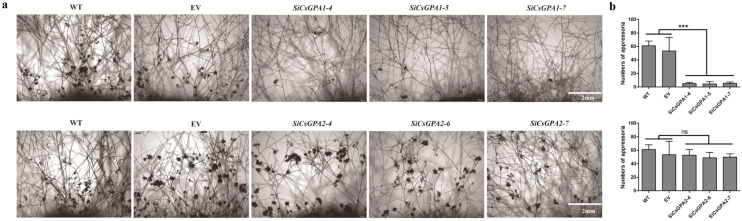
Compound appressoria formation in WT, EV, and gene-silenced strains (*CsGPA1* and *CsGPA2*). (**a**) Morphological features of appressoria were observed by bright field microscopy. The fresh agar plugs were inoculated on glass slides for 24 h. (**b**) The numbers of compound appressoria in WT, EV, and gene-silenced strains (*CsGPA1* and *CsGPA2*). All treatments were replicated three times. The data represent the means ± SD of three independent replicates, and the differences between the mutants and the controls or EV were analyzed by one-way ANOVA followed by Duncan’s multiple range test. Note: *** *p* < 0.001; ns, no significant difference at *p* < 0.05.

To further investigate the effects of *CsGPA1* and *CsGPA2* silencing on compound appressoria formation, hyphal morphology was observed using light microscopy at 24 hpi. The WT and EV strains formed abundant compound appressoria from vegetative hyphae by 24 hpi. Conversely, few compound appressoria were found in *CsGPA1* RNAi strains (Figure 3a). The *CsGPA2*-silenced strains had a similar number of compound appressoria to WT and EV (Figure 3a). The numbers of compound appressoria were significantly lower in *CsGPA1*-silenced strains compared with WT and EV; however, there was only a slight difference for *CsGPA2*-silenced strains (Figure 3b).

### 3.4. CsGPA1 Negatively Regulates Tolerance to Osmotic and Oxidative Stress but Is Dispensable for Extracellular Laccase and Peroxidase Activities

**Figure 4:**
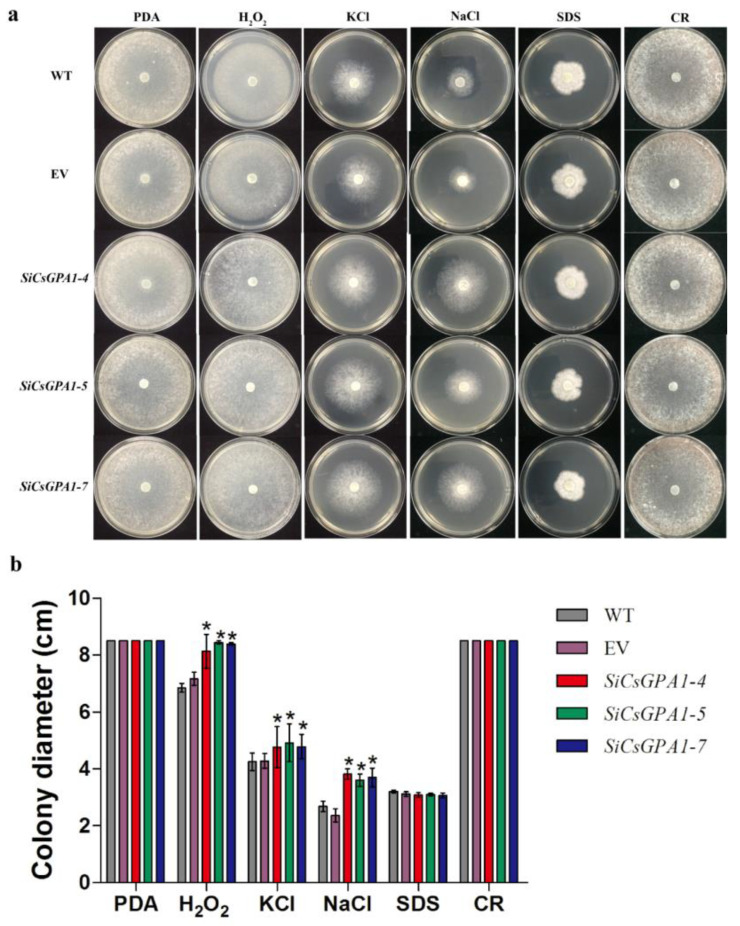
Silencing of *CsGPA1* improved the tolerance to osmotic and oxidative stresses: (**a**) colony morphology on PDA plates supplemented with different stress reagents for 48 h; (**b**) mycelial growth rate of the WT, EV, and *CsGPA1*-silenced strains on PDA supplemented with different stress reagents for 48 h. The data represent the means ± SD of three independent replicates, and the differences between the mutants and the controls or EV were analyzed by one-way ANOVA followed by Duncan’s multiple range test. Note: * *p* < 0.05.

To investigate the roles of *CsGPA1* and *CsGPA2* in adaptation to various stresses, the growth rates of WT, EV, and *CsGPA1* and *CsGPA2* RNAi strains on CM medium supplemented with the five stress agents were compared (Figure 4a, Figure S5). The *CsGPA1* RNAi strains grew faster than the WT and EV on plates supplemented with H_2_O_2_, KCl, or NaCl, but showed no differences in growth with SDS or CR (Figure 4b). Moreover, *CsGPA1* silencing did not change the activities of extracellular laccase or peroxidase (Figures S6 and S7). However, the growth of *CsGPA2* RNAi strains on CM supplemented with these five stress agents showed no obvious changes compared with WT and EV, although had slightly higher extracellular laccase and peroxidase activities (Figures S6 and S7).

### 3.5. Silencing CsGPA1 Significantly Reduces C. shiraiana Virulence on Tobacco

**Figure 5:**
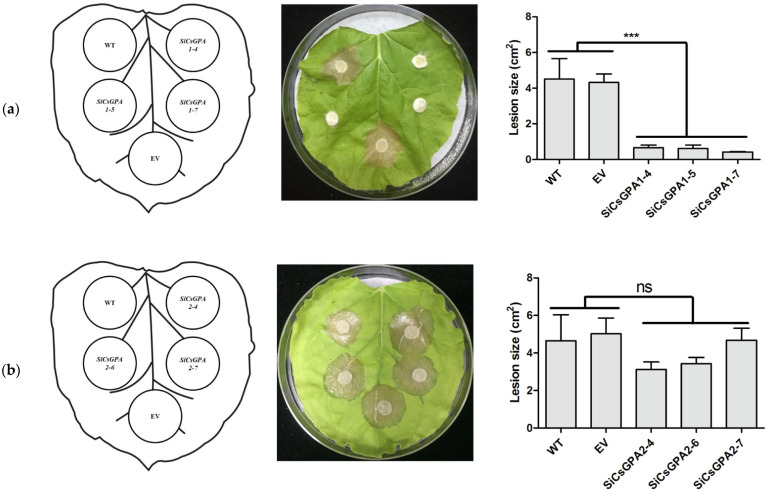
Virulence of WT, EV, and *CsGPA1*- and *CsGPA2*-silenced strains on tobacco leaves: (**a**) virulence and lesion areas of *CsGPA1* mutants on tobacco leaves; (**b**) virulence and lesion areas of *CsGPA2* mutants on tobacco leaves. The data represent the means ± SD of three independent replicates, and the differences between the mutants and the controls or EV were analyzed by one-way ANOVA followed by Duncan’s multiple range test. Note: *** significant difference at *p* < 0.001; ns, no significant difference at *p* < 0.05.

To analyze the roles of *CsGPA1* and *CsGPA2* in pathogenesis, virulence was evaluated by inoculation on *N. benthamiana* leaves. There were significantly less necrotic lesions for *CsGPA1*-silenced strains than WT and EV strains (Figure 5a). The *CsGPA2* RNAi strains also showed a slight non-significant reduction in virulence (Figure 5b).

### 3.6. Silencing CsGPA1 and CsGPA2 Downregulates Expression of cAMP and MAPK Signaling Genes

**Figure 6:**
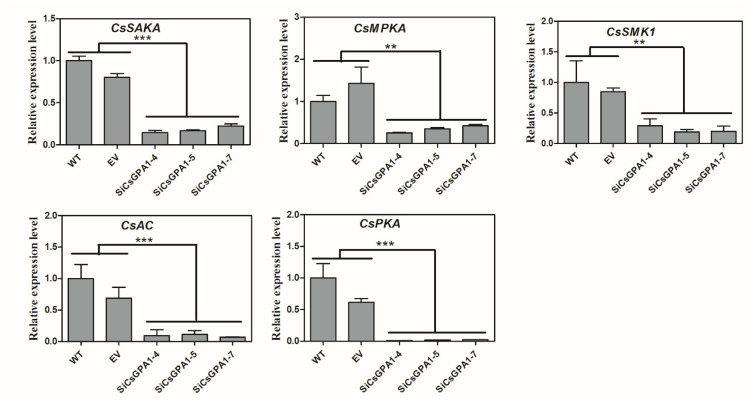
Relative expression levels of genes involved in cAMP/PKA and MAPK pathways in WT, EV, and CsGPA1-silenced strains. The data represent the means ± SD of three independent replicates, and the differences between the mutants and the controls or EV were analyzed by one-way ANOVA followed by Duncan’s multiple range test. Note: ** *p* < 0.01, *** *p* < 0.001.

Expression levels of three MAPK signaling genes (*CsSAKA*, *CsMPKA*, and *CsSMK1*) and two cAMP signaling related genes (*CsAC* and *CsPKA*) in *CsGPA1* RNAi strains showed significant downregulation compared with WT and EV strains (Figure 6). Furthermore, similar decreases in expression levels of these genes were found in *CsGPA2*-silenced strains (Figure S8).

### 3.7. CsGPA1 Silencing by Plant-Mediated RNAi Improves Plant Resistance to C. shiraiana

**Figure 7:**
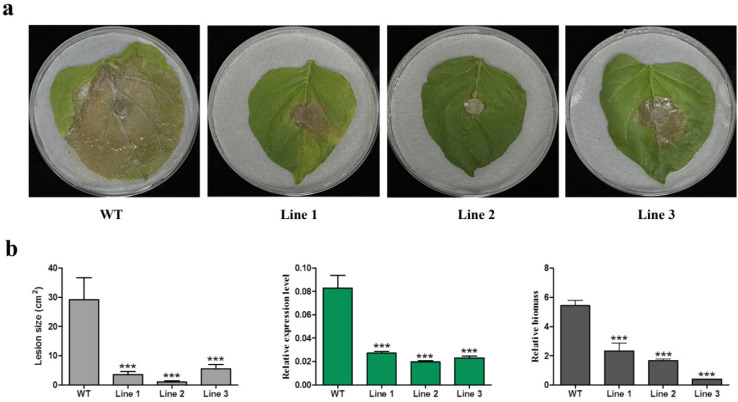
Host-induced gene silencing of *CsGPA1* improved the tobacco resistance against *C. shiraiana*: (**a**) disease symptoms on *N. benthamiana* leaves at 48 hpi; (**b**) lesion size (cm^2^), relative gene expression of *CsGPA1* and relative biomass in transgenic and WT plants. The data represent the means ± SD of three independent replicates, and the differences between the mutants and the controls or EV were analyzed by one-way ANOVA followed by Duncan’s multiple range test. Note: *** *p* < 0.001.

Silencing of *CsGPA1* significantly reduced the *C. shiraiana* virulence, so *CsGPA1* was used as the target to generate HIGS transgenic tobacco. A total of seven independent transgenic tobacco lines were obtained and three lines were used for a pathogen infection experiment. The transgenic tobacco lines all showed enhanced resistance to *C. shiraiana* to different extents (Figure 7a), mainly apparent as fewer necrotic lesions and lower relative pathogen biomass compared with WT tobacco (Figure 7b). Meanwhile, the level of *CsGPA1* expression in hypha inoculated on transgenic tobacco leaves was significantly reduced (Figure 7b).

**Figure 8:**
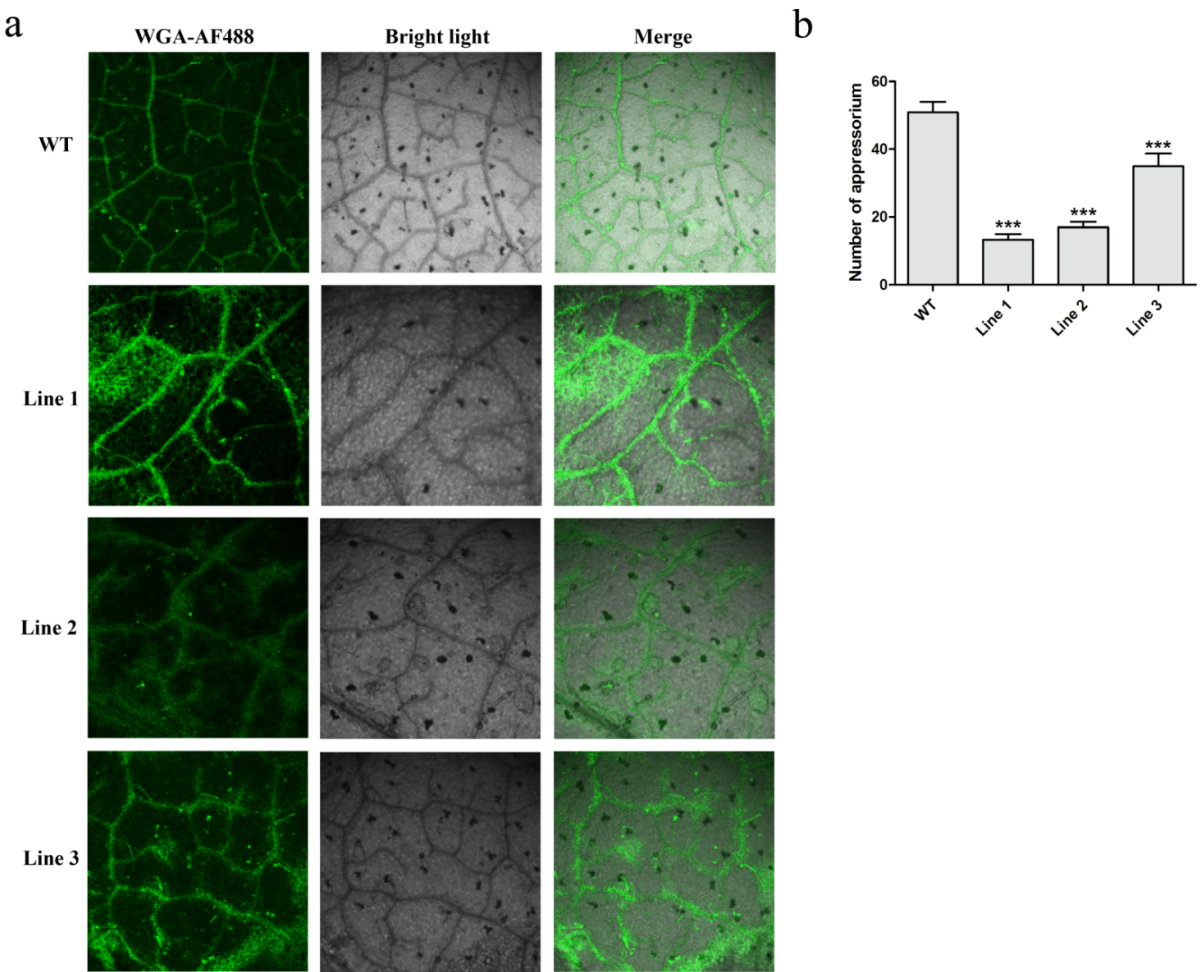
Histological observation of fungal growth in WT and transgenic tobacco leaves: (**a**) fungal growth at 12 hpi in WT and HIGS of *CsGPA1* plants; (**b**) significant decrease in the number of appressoria. The data represent the means ± SD of three independent replicates, and the differences between the mutants and the controls or EV were analyzed by one-way ANOVA followed by Duncan’s multiple range test. Note: *** *p* < 0.001.

To further research the effects of HIGS tobacco on *C. shiraiana* development and infection, the morphology of hyphae inoculated on transgenic tobacco leaves was observed by microscope. The number of infection hyphae, indicating pathogenicity, was significantly reduced compared with WT tobacco (Figure 8a). In addition, significantly fewer compound appressoria were formed on transgenic tobacco leaves compared to WT plants, indicating that transgenic tobacco reduced the virulence of *C. shiraiana* (Figure 8b).

## 4. Discussion

Heterotrimeric G-protein signaling pathways play important roles in the regulation of fungal growth, appressoria formation, and pathogenicity [13]. The G protein α subunits are the key component of trimeric G protein signals, which serve as molecular switches to regulate a series of cellular processes [18,20]. In this study, *CsGPA1* and *CsGPA2* were mainly expressed during sclerotia formation and late infection stages, suggesting that they were involved in the regulation of sclerotia formation and infection. Sclerotia and appressoria formation is vital for fungal spread and infection. In *C. shiraiana*, a maximum of 15 apothecia can germinate from a single sclerotium, and each apothecia measuring approximately 1.5 cm in diameter can release 5.6 × 10^7^ to 6.3 × 10^7^ ascospores [8]. In the filamentous fungus *S. sclerotiorum*, the *Shk1-*, *SCD1-*, and *THR1*-deletion mutants show significantly reduced melanin biosynthesis and sclerotia formation [43,44]. In this study, the *CsGPA1-* and *CsGPA2*-silenced mutants only formed sclerotia on the edge of the plate in lower numbers, suggesting that *CsGPA1* and *CsGPA2* were involved in the regulation of sclerotia formation via reduced melanin biosynthesis.

The functions of G protein α subunits in regulating vegetative growth vary in different fungal species. In *M. robertsii*, *MrGpa1* deletion did not affect vegetative growth on PDA medium [18]. In *Monascus ruber* M7, the *Mga2* and *Mga3* knockout strains also showed no significant effects on vegetative growth [20]. However, in *A. fumigatus*, *gpaB* and *ganA* deletion resulted in decreased colony growth and increased growth of the *gpaA* deletion strain in minimal medium [19]. In this study, the findings suggested that *CsGPA1* and *CsGPA2* were not involved in vegetative growth. Therefore, the function of G protein α subunits is not completely conserved in fungal species.

In previous studies, G protein α subunits showed important roles in the regulation of sensitivity to stresses. For instance, *MrGpa1* deletion strains of *M. robertsii* showed an increased tolerance to H_2_O_2_ [18]. In *Penicillium camemberti*, overexpression of *pga1*^G42R^ reduced osmotic stress tolerance to 1.5 M NaCl and 1.5 M KCl [45]. In this research, similar results were obtained in *CsGPA1*-silenced strains, indicating that *CsGPA1* negatively regulated resistance to osmotic and oxidative stresses. Laccase activity is involved in infection by some fungi [46]. Several lower-virulence mutants of *M. oryzae* also showed reduced laccase and peroxidase activities [47,48,49]. In our study, *CsGPA2* silencing slightly improved laccase and peroxidase activities. However, *CsGPA1*-silenced strains had reduced virulence but normal laccase and peroxidase activity, indicating that *CsGPA1*-regulated pathogenicity may be independent of laccase and peroxidase in *C. shiraiana*.

In previous research, the G protein α subunit was shown to play an important role in appressoria formation. In *M. grisea*, disruption of *mag*B significantly reduced appressoria formation, and a similar result was found in *M. robertsii* for *MrGPA1* deletion strains [18,50]. The cAMP/PKA and MAP kinase signaling are important signal transduction cascades in regulating fungal appressoria formation and pathogenicity [51]. In *M. robertsii*, *Ste11-*, *Ste7-*, and *Fus3*-deletion strains showed significant decreases in appressoria formation and pathogenicity [52]. Similarly, the silencing of *PsMPK7* from *Phytophthora sojae* reduced its oospore production and pathogenicity to soybean [53]. In addition to MAPK signaling, cAMP/PKA signal transduction has been well studied in the pathogenesis of several fungi, such as *MaPKA1* in *M. anisopliae* and *MAC1* from *M. grisea*; these genes seem to be involved in the regulation of appressoria formation and pathogenicity [54,55]. Furthermore, Gα4 regulated developmental morphogenesis in *Dictyostelium* by interacting with the MAPK ERK2 while the *GpaB* mutant had reduced pathogenicity by regulating cAMP/PKA signaling in *Aspergillus flavus* [56,57]. Therefore, G protein α subunits are involved in cAMP/PKA and MAP kinase signaling. In this study, silencing of *CsGPA1* significantly reduced appressoria formation and virulence. Meanwhile, the expression levels of cAMP/PKA- and MAPK-signaling-related genes in *CsGPA1* mutants were significantly decreased compared with WT. These results demonstrate that *CsGPA1* is involved in the cAMP/PKA and MAPK signal transduction pathways in *C. shiraiana*, which control appressoria formation and pathogenicity.

*Ciboria shiraiana* is part of the Sclerotiniaceae family, and is considered to have a narrow host range due to its low amounts of secreted effector proteins [35]. Mulberry is a woody plant. It is very difficult to obtain transgenic mulberry because of its longer growth cycle and the lack of a stable transformation system. *Nicotiana benthamiana* is widely used as a model plant in research on biotic stress owing to its short growth period [58]. Previous studies have shown that *C. shiraiana* could infect *N. benthamiana* leaves rapidly and can serve as a model for performing infection tests [58]. Studies have also revealed that *C. shiraiana* can cause disease in tomato and rapeseed [35,59]. It is urgent to develop an economical, effective, and environmentally friendly method to control this disease. In some biotrophic and necrotrophic fungi, HIGS has been demonstrated as a new strategy to reveal gene function [29,32]. Cross-kingdom RNAi depends on the efficiency of fungal take-up of environmental double-stranded RNAs [60,61,62]. Both *B. cinerea* and *S. sclerotiorum* are also members of the Sclerotiniaceae, and can take-up environmental RNA with high efficiency, indicating that *C. shiraiana* may also be suited to HIGS [60]. Our results proved that *CsGPA1* is an efficient target to increase the tolerance to *C. shiraiana*.

## 5. Conclusions

In conclusion, two G protein α subunit genes were isolated from *C. shiraiana*. Silencing *CsGPA1* and *CsGPA2* did not affect vegetative growth but reduced sclerotia formation. Moreover, *CsGPA1*-silenced strains showed reduced appressoria formation and virulence and improved tolerance to osmotic and oxidative stresses. Expressing the double-stranded RNA targeted to *CsGPA1* in tobacco improved the tolerance to *C. shiraiana*. These data demonstrate that *CsGPA1* is required for full virulence of *C. shiraiana* and is an efficient target to improve tolerance to *C. shiraiana* using HIGS technology.

## Data Availability

Not applicable.

## Supplementary Materials

The following are available online at https://www.mdpi.com/article/10.3390/jof7121053/s1: Figure S1. Schematic diagram of RNAi (a) and HIGS (b) plasmids constructs. Figure S2: Gene features and phylogenetic analysis of CsGPA1 and CsGPA2. Figure S3: Generation of *CsGPA1-* and *CsGPA2*-silenced strains. Figure S4: Hyphal growth in wild-type (WT), empty vector (EV), and gene-silenced strains (*CsGPA1* and *CsGPA2*). Figure S5: Silencing of *CsGPA2* did not affect the tolerance to osmotic, cell wall integrity, and oxidative stresses. Figure S6: Measurement of extracellular laccase activities. Figure S7: Measurement of extracellular peroxidase activity. Figure S8: Relative expression levels of genes involved in cAMP/PKA and MAPK pathways in WT, EV, and *CsGPA2*-silenced strains. Table S1: Primers used in this experiment.
