# Supplementary material for: Host-Induced Gene Silencing of a G Protein α Subunit Gene CsGpa1 Involved in Pathogen Appressoria Formation and Virulence Improves Tobacco Resistance to Ciboria shiraiana
Source: J Fungi (Basel). 2021 Dec 8;7(12):1053. doi: 10.3390/jof7121053 (PMC8709418; doi:10.3390/jof7121053)
Supplement: Supplementary file 1 [file jof-07-01053-s001.zip › jof-1472174-supplementary.pdf]

Journal of *Fungi*

MDPI

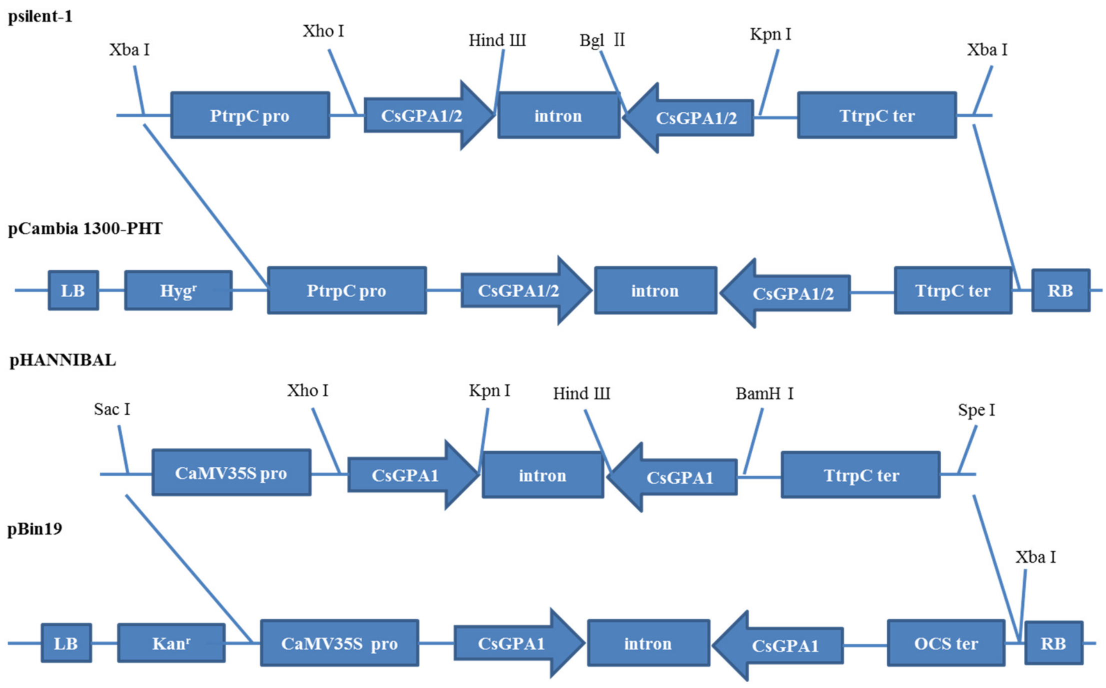

Figure S1. Schematic diagram of RNAi (a) and HIGS (b) plasmids constructs.

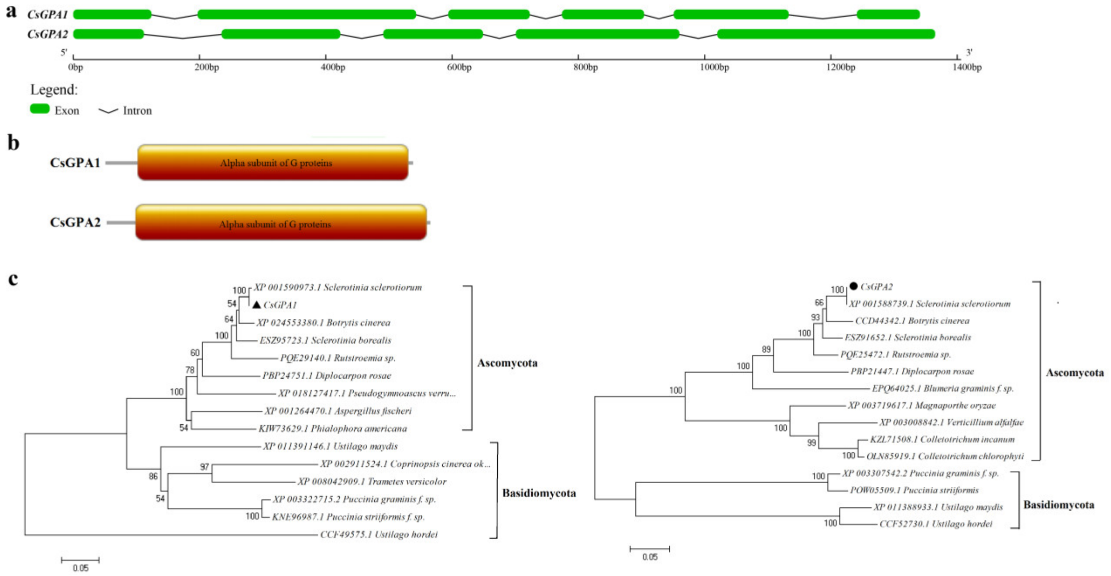

Figure S2. Gene features and phylogenetic analysis of CsGPA1 and CsGPA2. (a) Intron/exon distribution of *CsGPA1* and *CsGPA2* genes. (b) Prediction of functional domain of CsGPA1 and CsGPA2. (c) Phylogenetic analysis of CsGPA1 and CsGPA2. The phylogenetic tree was constructed with MEGA 4.0 using a bootstrap test of phylogeny with a minimum evolution test and default parameters, and the genes studied are marked with black triangle and circle.

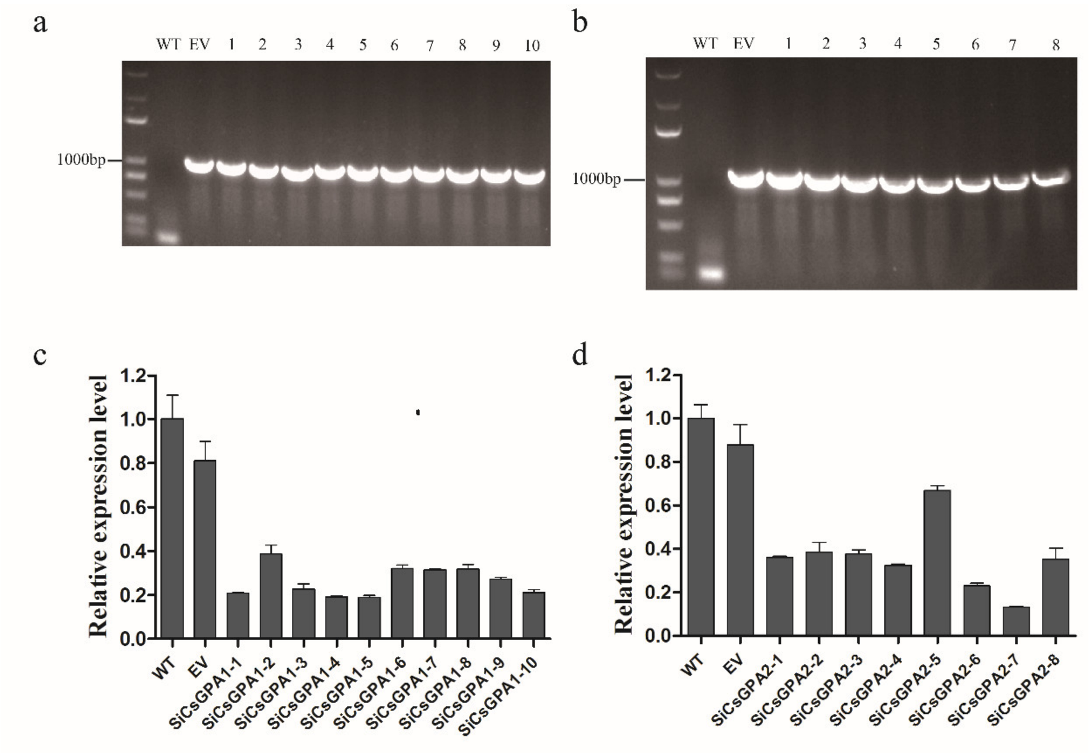

Figure S3. Generation of *CsGPA1*- and *CsGPA2*-silenced strains. (a) and (b) Amplification of the hygromycin gene from the genomes DNA of WT, EV, and silenced strains (1-10 in (a): *SiCsGPA1-1* to *SiCsGPA1-10*; 1-8 in (b): *SiCsGPA2-1* to *SiCsGPA2-8*). (c) *CsGPA1* and (d) *CsGPA2* expression levels in wild-type (WT), empty vector (EV), and silenced mutants.

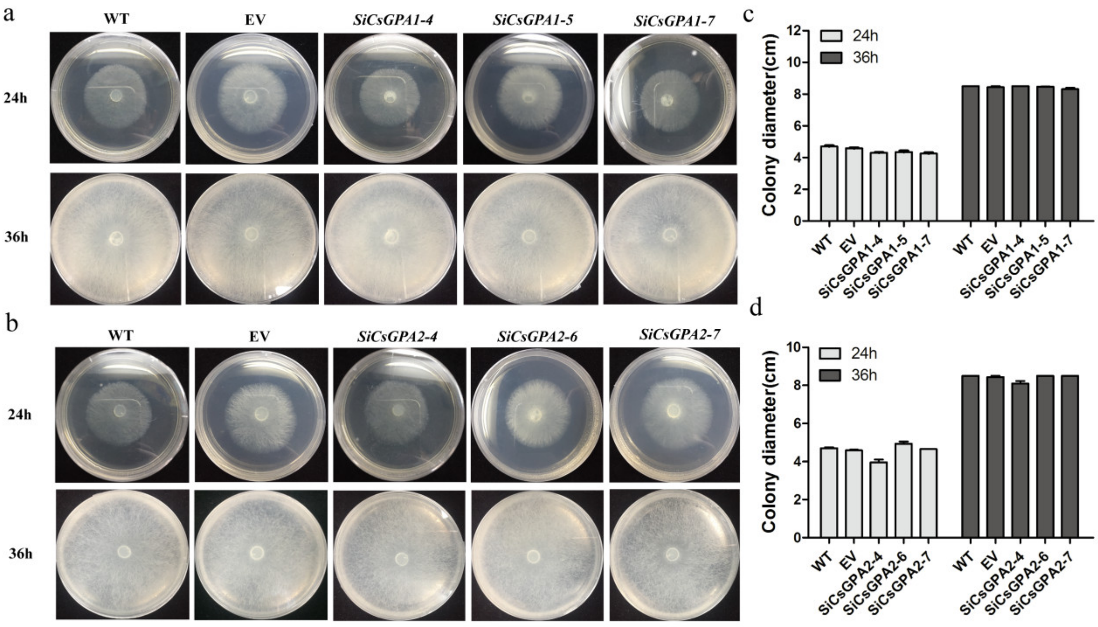

Figure S4. Hyphal growth in wild-type (WT), empty vector (EV), and the gene-silenced strains (*CsGPA1* and *CsGPA2*). Colony morphology of *CsGPA1* (a) and *CsGPA2* (b) mutants cultured on PDA at 24 and 36 h. Colony diameter of *CsGPA1* (c) and *CsGPA2* (d) mutants cultured on PDA at 24 and 36 h. The data represent the means ± SD of three independent replicates, and the differences between the mutants and the controls or EV were analyzed by one-way ANOVA followed by Duncan's multiple range test

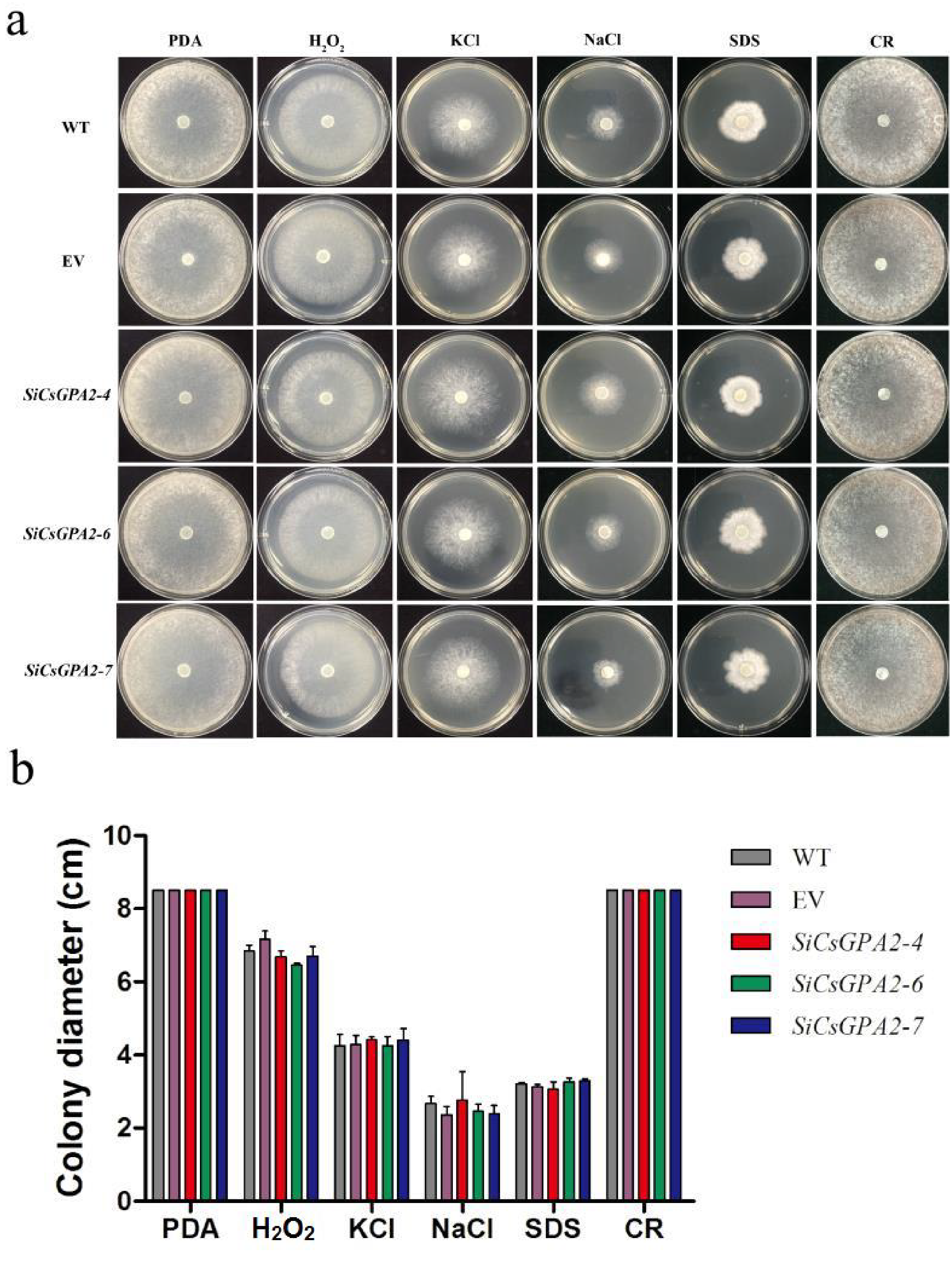

Figure S5. Silencing of *CsGPA2* did not affect the tolerance to osmotic, cell wall integrity, and oxidative stresses. (a) Colony morphology on PDA plates supplemented with different stress reagents for 48 h. (b) Mycelial growth rate of the WT, EV, and *CsGPA2*-silenced strains on PDA supplemented with different stress reagent for 48 h. * difference significant at $p < 0.05$. .

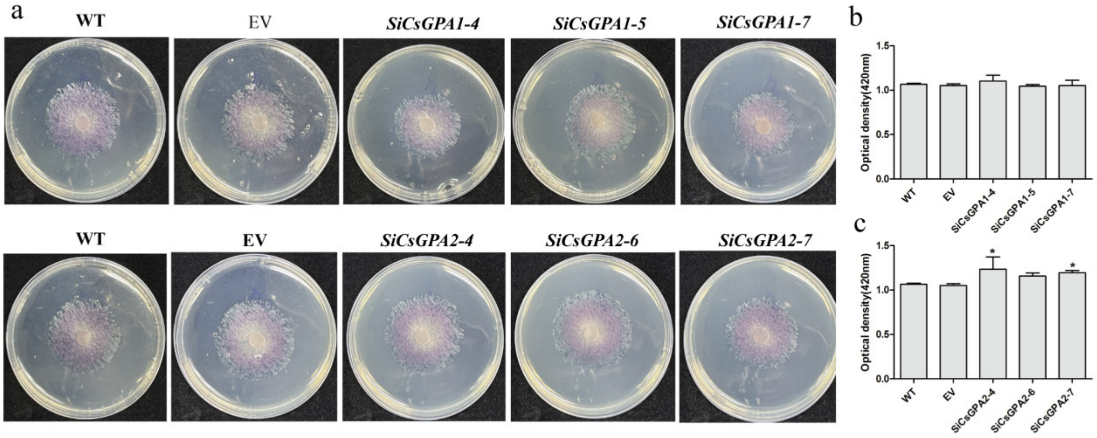

Figure S6. Measurement of extracellular laccase activities. (a) Laccase activity on CM agar plates containing 0.2 mM ABTS. Discolorations photographed at 48 hpi. (b) Laccase activity measured by ABTS oxidizing test. The absorbance was evaluated at 420 nm. The data represent the means ± SD of three independent replicates, and the differences between the mutants and the controls or EV were analyzed by one-way ANOVA followed by Duncan's multiple range test

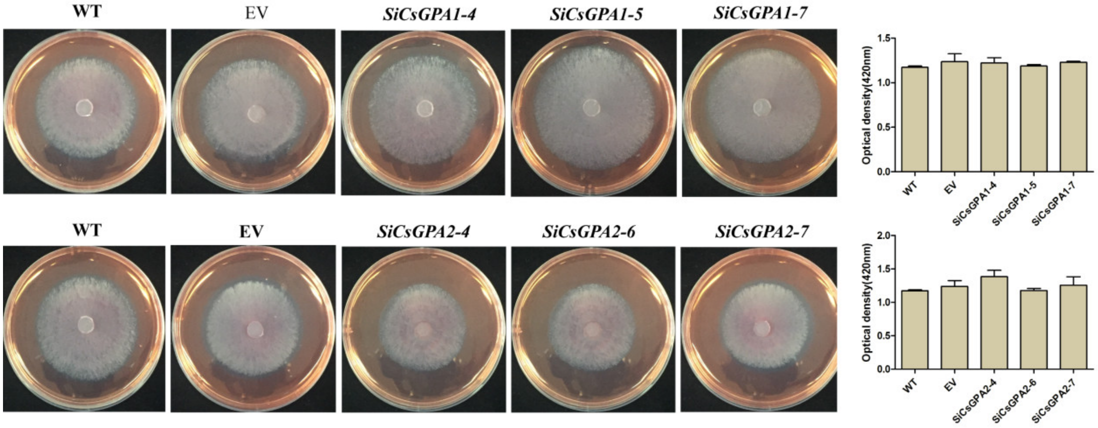

Figure S7. Measurement of extracellular peroxidase activity. (a) Peroxidases activity on CM agar plates containing 200 mg/l Congo Red. Discolorations were photographed at 48 hpi. (b) Peroxidase activity measured by ABTS oxidizing test supplemented with 3mM H_2O_2. The absorbance was evaluated at 420 nm. The data represent the means ± SD of three independent replicates, and the differences between the mutants and the controls or EV were analyzed by one-way ANOVA followed by Duncan's multiple range test.

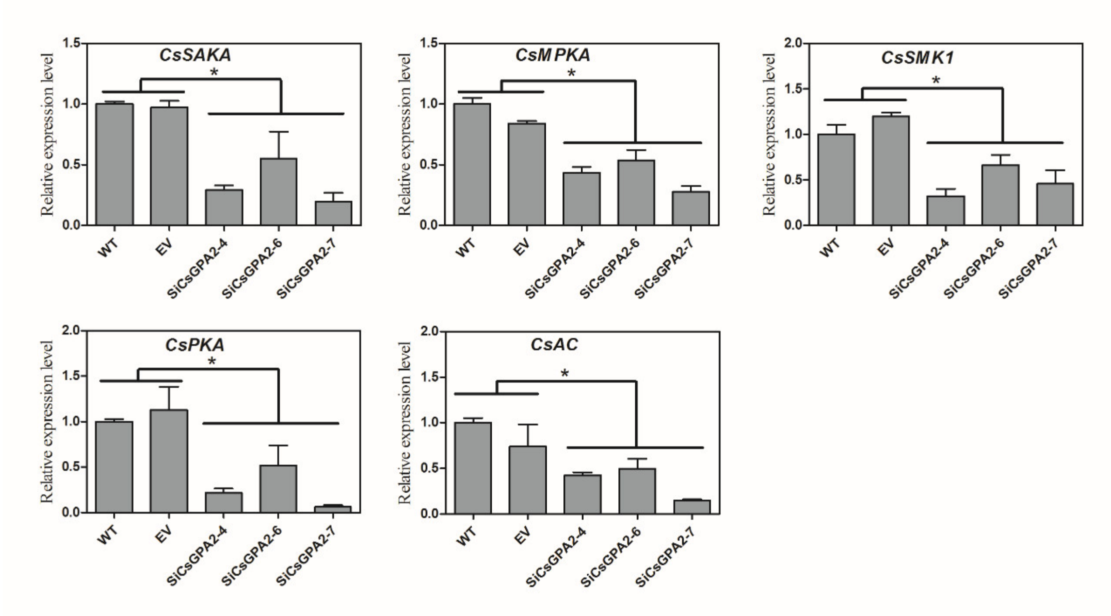

Figure S8. Relative expression levels of genes involved in cAMP/PKA and MAPK pathway in WT, EV, and *CsGPA2*-silenced strains. "*" difference significant at $p < 0.05$.

Table S1. Primers used in this experiment.

Primer	Primer sequence (5'-3')	Usage
CsGPA1-F	ATGTTTGATGTGGGTGGACA	Cloning of *CsGPA1*
CsGPA1-R	TTAAAGTATGCCCGAGTCTT	
CsGPA2-F	ATGTGTTTCGGCAGCAGGGA	Cloning of *CsGPA2*
CsGPA2-R	TCAAAGGATCAACTTTTGAA	
SiCsGPA1-F (*Xho* Ⅰ)	CCGCTCGAGGAAGCTGGAAGGATAGCAGC	RNAi vector construction
SiCsGPA1-R (*Hind*Ⅲ)	CCCAAGCTTAATACTGGTTCGCATAAACC	
SiCsGPA1-F1 (*Kpn* Ⅰ)	CGGGGTACCGAAGCTGGAAGGATAGCAGC	
SiCsGPA1-R1 (*Bgl* Ⅱ)	GGAAGATCTAATACTGGTTCGCATAAACC	
SiCsGPA2-F (*Xho* Ⅰ)	CCGCTCGAGACAGAGGGAATGAGTTTGCT	
SiCsGPA2-R (*Hind*Ⅲ)	CCCAAGCTTCCATCCTTGTCTTCAGCTAG	
SiCsGPA2-F1 (*Kpn* Ⅰ)	CGGGGTACCACAGAGGGAATGAGTTTGCT	
SiCsGPA2-R1 (*Bgl* Ⅱ)	GGAAGATCTCCATCCTTGTCTTCAGCTAG	
Hyb-F	ATGAAAAAGCCTGAACTCAC	
Hyb-R	CTATTCCTTTGCCCTCGGAC	
dsCsGPA1-F (*Xho* Ⅰ)	CCGCTCGAGGAAGCTGGAAGGATAGCAGC	HIGS vector construction
dsCsGPA1-R (*Kpn* Ⅰ)	CGGGGTACCAATACTGGTTCGCATAAACC	
dsCsGPA1-F1 (*BamH* Ⅰ)	CGCGGATCCGAAGCTGGAAGGATAGCAGC	
dsCsGPA1-R1 (*Hind*Ⅲ)	CCCAAGCTTAATACTGGTTCGCATAAACC	
Kan-F	GGTGCCCTGAATGAACTGCA	
Kan-R	GGTAGCCAACGCTATGTCCT	
NbActin-F	TCACAGAAGCTCCTCCTAATCCA	

NbActin-R	GAGGGAAAGAACAGCCTGAATG	Reference gene of *N. benthamiana*
Tubulin-F<br>*Tubulin*-R	TTGGATTTGCTCCTTTGACCAG<br>AGCGGCCATCATGTTCTTAGG	Reference gene of *C. shiraiana*
CsGPA1-QF<br>*CsGPA1*-QR<br>*CsGPA2*-QF<br>*CsGPA2*-QR	GCTCATCTGAATTTATACCCACACC<br>CCCGAGTCTTTGAGAGCGTT<br>TCGACCACTCGAACCAATCC<br>GTTGGGACGAACTCCTTGCT	qRT-PCR
